# Editorial: Translational applications of neuroimaging, volume II

**DOI:** 10.3389/fnins.2026.1809421

**Published:** 2026-03-26

**Authors:** Amelie Haugg, David M. A. Mehler, Stavros Skouras

**Affiliations:** 1 Department of Child and Adolescent Psychiatry, Psychiatric University Hospital, University of Zürich, Zürich, Switzerland; 2 Department of Psychiatry, Psychotherapy and Psychosomatics, Faculty of Medicine, Rheinisch–Westfälische Technische Hochschule (RWTH) Aachen University, Aachen, Germany; 3 Institute for Translational Psychiatry, University of Münster, Münster, Germany; 4 Cardiff University Brain Research Imaging Centre (CUBRIC), School of Psychology, Cardiff University, Cardiff, United Kingdom; 5 Department of Fundamental Neurosciences, Faculty of Medicine, University of Geneva, Geneva, Switzerland

**Keywords:** EEG, MRI, multimodal neuroimaging, neuroimaging, neurology, psychiatry, translational neuroimaging, neurofeedback

Clinical translation of neuroimaging methods remains challenging. Following our first volume on Translational Applications of Neuroimaging (Haugg et al., 2024), the seven articles in this second volume present further approaches to bridging the gap between brain research methods and clinical tools. Here, a common theme emerges across various disorders [Parkinson's disease, major depressive disorder (MDD), post-traumatic stress disorder (PTSD), and schizophrenia], as well as across various neuroimaging methodologies [multi-modal Magnetic Resonance Imaging (MRI), Electroencephalography (EEG), functional MRI (fMRI), and neurofeedback training (NFT) where participants self-regulate brain function based on real-time neuroimaging information]. While novel advances in technology for diagnosis and intervention are promising, methodological and implementation barriers remain an obstacle for clinical applications.

Balßuweit et al. used multi-modal MRI and machine learning to classify cognitive performance in patients with Parkinson's disease. Their study demonstrates the potential of multi-modal neuroimaging for applications enabling the earlier detection of cognitive deficits and improving intervention and care planning for more than 10 million patients living with Parkinson's disease worldwide. Ultimately, the clinical translation of such a method will depend on future validation success across multi-center cohorts and a widespread adoption of harmonized acquisition protocols.

Mähler and Reichenbach conducted a systematic review of resting-state EEG studies with MDD patients to discover that the substantial heterogeneity in study population characteristics is an often neglected source of inconsistency that impedes the development of effective neuromarkers. They emphasized the need for establishing deeper profiling practices, beyond the simple patient vs. control grouping, as well as the need for characterizing samples and describing analyses in more detail, to enable better comparisons and integration of findings across studies. These points are

generalizable beyond MDD, as improving the standards of precision and methodological rigor is necessary for the field of translational neuroimaging to reach its fruition.

In a systematic review and meta-analysis of EEG and fMRI NFT in PTSD, Berman et al. found moderate to large effect sizes regarding symptom reductions, based on five included studies. However, the only two of those studies that also included sham NFT did not detect any superiority for the intervention. Furthermore, no definitive evidence exists regarding specific neural effects of NFT in PTSD. The authors conclude that, across the included studies, the risk of bias, the level of imprecision, and the possibility for conflicts of interest were relatively high, despite the fact that two-thirds of the studies were pre-registered. This underscores a broader pattern in translational neuroimaging where promising early findings coexist with methodological limitations and limited mechanistic clarity.

Complementing these findings, Askovic et al. performed a feasibility study to test the effects of EEG NFT combined with trauma counseling in 47 refugees with chronic, treatment-resistant PTSD over a median of 26 sessions across 7 months. The study did not include a measured control group and was limited to historical control data of 107 healthy participants retrieved from an EEG database. However, it provides proof of concept by showing that approximately half of the study's participants exhibited a clinically meaningful level of PTSD symptom reduction, alongside normalized ERPs, following NFT. Outcome reporting bias, however, cannot be excluded due to a lack of study pre-registration. The hypothesized mechanistic link will require investigation in a larger, pre-registered randomized controlled trial.

Another two systematic reviews examined functional disruptions and NFT intervention effects, respectively, in schizophrenia patients. The systematic review by Jensen et al. utilized a unified nomenclature based on the NeuroMark atlas across fMRI resting-state studies to compare schizophrenia patients with healthy controls. They observed that schizophrenia was most consistently characterized by functional connectivity disruptions in the cerebellum, thalamus, and sensorimotor cortex. Duan et al. performed a systematic review and meta-analysis of 14 RCTs, including 1,371 patients in total, to investigate the effect of complementing the standard pharmacological treatment of schizophrenia with EEG-based NFT. Results show there is a large effect for the combined intervention, with both positive and negative schizophrenia symptoms improving significantly, though more strongly concerning negative symptoms. Further, the authors report dose effects of improvement associated with patient age and disease duration, as well as the mode, period, and frequency of EEG NFT. The greatest benefits were observed when NFT lasted at least 8 weeks and occurred at least four times per week, especially with protocols targeting sensorimotor rhythm and beta waves. However, these findings must also be considered with caution, as the authors warn of the risk of bias due to a lack of study pre-registrations as well as limited sample sizes, noting that larger multicentre studies using standardized NFT protocols are required to confirm the effectiveness of the approach and elucidate the underpinning neural mechanisms.

Lastly, Sayal et al. conducted a systematic review of music-based neurofeedback studies to further explore the potential of music as a tool for self-regulation of brain activity. The authors summarized promising implementations that were typically motivated by music's emotion-evoking properties and its potential for emotion regulation, but criticized the lack of mechanistic explanations, standardized outcome measures, and study pre-registration. Once more, these limitations mirror those identified in other NFT domains.

In conclusion, the articles comprising this volume highlight promises for a number of highly impactful translational applications of neuroimaging, while at the same time they point to specific methodological shortcomings that need to be overcome for the field to fulfill its potential. Specifically, the seven contributions in this Special Topic converge on two interrelated translational bottlenecks. First, heterogeneity without sufficient harmonization limits the reproducibility of results. Second, symptom improvements and diagnostic advances are often reported without mechanistic validation or evidence of neural specificity. Thus, the need for standardized practices, methodological rigor, deeper profiling, and detailed characterization is fundamental to catalyse future progress. As a priority, comprehensive study pre-registrations or Registered Reports, which ensure peer reviewing of the study protocol and acceptance in principle before data collection and analysis, are a readily implementable measure that should be considered essential (Lakens et al., 2024). If these requirements are met, the promise of translational neuroimaging may move closer to sustained clinical impact rather than remaining a compelling research objective.
